# Overexpression of *SmANS* Enhances Anthocyanin Accumulation and Alters Phenolic Acids Content in *Salvia miltiorrhiza* and *Salvia miltiorrhiza* Bge f. *alba* Plantlets

**DOI:** 10.3390/ijms20092225

**Published:** 2019-05-06

**Authors:** Hongyan Li, Jingling Liu, Tianlin Pei, Zhenqing Bai, Ruilian Han, Zongsuo Liang

**Affiliations:** 1 College of Life Sciences, Northwest A& F University, Yangling 712100, China; lihongyan2019@126.com (H.L.); jinglingliu-sm@nwsuaf.edu.cn (J.L.); tianlinpei@163.com (T.P.); shanxibzq@163.com (Z.B.); 2 School of Civil Engineering and Architecture, Zhejiang Sci-Tech University, Hangzhou 310018, China; 3 College of Life Sciences and Medicine, Zhejiang Sci-Tech University, Hangzhou 310018, China

**Keywords:** *Salvia miltiorrhiza*, *SmANS*, *Salvia miltiorrhiza* Bge f. *alba*, flavonoids, anthocyanin, phenolic acids

## Abstract

Flavonoids play multiple roles in plant coloration and stress resistance and are closely associated with human health. Flavonoids and non-flavonoids (such as phenolic acids) are produced via the phenylpropanoid-derived pathway. Anthocyanidin synthase (ANS) catalyzes the synthesis of anthocyanins from leucoanthocyanidin in the flavonoids branched pathway. In this study, *SmANS* from *Salvia miltiorrhiza* was cloned and mainly localized in the endoplasmic reticulum (ER), plastids, Golgi, plasma membrane, and nucleus of tobacco epidermal cells, and was most highly expressed in purple petals in *S. miltiorrhiza*, whereas it showed almost no expression in white petals, green calyxes, and pistils in *S. miltiorrhiza* Bge f. *alba*. Overexpressed *SmANS* enhanced anthocyanin accumulation but reduced salvianolic acid B (SAB) and rosmarinic acid (RA) biosynthesis in *S. miltiorrhiza* and *S. miltiorrhiza* Bge f. *alba* plantlets, meanwhile, it restored the purple-red phenotype in *S. miltiorrhiza* Bge f. *alba*. These changes were due to reallocation of the metabolic flow, which was influenced by the *SmANS* gene. These findings indicate that *SmANS* not only plays a key role in anthocyanin accumulation in *S. miltiorrhiza*, but also acts as a “switch” for the coloration of *S. miltiorrhiza* Bge f. *alba*. This study provides baseline information for further research on flavonoids metabolism and improvement of anthocyanin or phenolic acid production by genetic engineering.

## 1. Introduction

*Salvia miltiorrhiza* Bunge (Danshen), which has purple flowers, is a traditional medicinal model plant with great medicinal and economic value, as well as an efficient genetic transformation system [1]. It has a white-flowered variant, named *S. miltiorrhiza* Bge f. *alba* (also called white-flowered Danshen in China) [2]. Although, the main effective compounds in Danshen medicinal materials are lipophilic tanshinones and hydrophilic phenolic acids, such as salvianolic acid B (SAB) and rosmarinic acid (RA) [3], abundant flavonoids components are distributed in their aerial parts [4]. However, these beneficial flavonoids are not well utilized because of the lack of awareness among people.

Flavonoids are also the major pigments responsible for the coloration of plant flowers, leaves, stems, fruits, and other organs [5]. They have been classified into different subgroups, including anthocyanin, proanthocyanidins, flavones, flavonols, flavanones, aurones, and isoflavones. Furthermore, they have various active biological roles in plant growth and development [6]. For instance, these flavonoids metabolites are involved in the color generation of plant tissues and organs, in attracting beneficial pollinators and symbionts, and in defending plants against biotic and abiotic stresses, such as insects, pathogens microbes, drought, cold, UV radiation, auxins, ABA, jasmonates, and so on [7,8,9,10]. In addition, flavonoids have a curative effect on many types of cancer, se-nilism, neuronal diseases, cardiovascular illnesses, diabetes, inflammation, and others diseases, and have been widely used for the prevention and treatment of diseases, as well as protection of human health [11].

**Figure 1:**
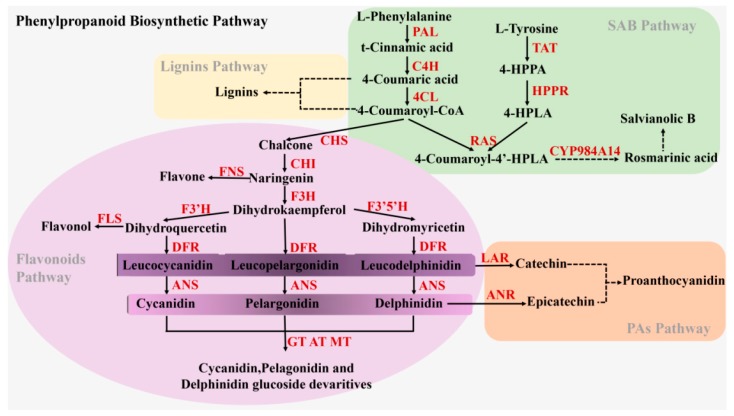
The metabolic pathways of the flavonoids and phenolic acids in *Salvia miltiorrhiza*. Solid arrows and dashed arrows represent single-step reactions and multiple steps reactions, respectively. Multiple enzymes are indicated in red text. Abbreviations: PAL, phenylalanine ammonia-lyase; C4H, cinnamic acid 4-hydroxylase; 4CL, hydroxycinnamate coenzyme A ligase; CHS, chalcone synthase; CHI, chalcone isomerase; FNSII, Flavone synthase; F3H, flavanone 3-hydroxylase; F3’H, flavonoid 3′-hydroxylase; F3′5′H, flavonoid 3′5′-hydroxylase; DFR, dihydroflavonol reductase; ANS, anthocyanidin synthase; FLS, Flavonol synthase; GT, UDP-glucose: anthocyanidin/flavonol 3-*O*-glucosyltransferase; AT, acyltransferase; MT, methyltransferases; LAR, leucoanthocyanidin reductase; ANR, anthocyanidin reductase; TAT, tyrosine aminotransferase; HPPR, 4-hydroxyphenylpyruvate reductase; RAS, rosmarinic acid synthase; CYP98A14, a cytochrome P450-dependent monooxygenase.

Flavonoids and other non-flavonoids (phenolic acids and lignin) metabolites are produced via different branches of the phenylpropanoid biosynthetic pathway in *S. miltiorrhiza* (Figure 1) [12], and compete with each other for identical precursors (4-coumaroyl-CoA or 4-coumaric acid). The flavonoids branched pathway is controlled by a series of enzymes, such as chalcone synthase (CHS), chalcone isomerase (CHI), anthocyanidin synthase (ANS), and so on. In the phenolic acids branched pathway in *S. miltiorrhiza*, SAB biosynthesis has been thought to be derived from an RA precursor through two parallel pathways (phenylpropanoid-derived and tyrosine-derived pathways) [12]. Crosstalk of metabolic flow between flavonoids and other flavonoids pathways occurs regularly. In recent years, researchers have also focused on flavonoids biosynthesis because of the molecular and metabolic crosstalk. Based on the strategies of switching or reducing the synthesis of the flavonoids- or lignin-branched pathways, genetic engineering technology has been applied in *S. miltiorrhiza* to improve phenolic acids productions by the RNAi-mediated silencing of the *SmCHS* port enzyme gene in the flavonoids-branched pathway [13], co-suppression of two key endogenous enzymes (*SmCCR* and *SmCOMT*) in the lignin-branched pathway, plus ectopic expression of *AtPAP1* [12]. Thus, these are also efficient methods to enhance the accumulation of SAB and RA bioactive compounds by intervening with other genes besides of the phenolic acids branched pathway. The key genes and their regulated transcription factors (myeloblastosis, helix−loop−helix, and WD repeat protein) involved in flavonoids and non-flavonoids biosynthesis, including the phenylpropanoid biosynthetic pathway, have been studied and reported in numerous plants [9,14,15,16]. MYB transcription factors often have a regulatory effect on the phenylpropanoid pathway, and interact with bHLH and WD to form MBW complexes [17,18]. In *S. miltiorrhiza*, *SmTTG1* and *SmMYB36* have been found to be involved in regulating the accumulation of both phenolic acids and anthocyanin [19,20,21]. 

However, *ANS*, which is one of the key enzyme genes downstream of the flavonoids-branched pathway, and catalyzes the transformation of leucoanthocyanidin to colored anthocyanidin before the final glycosylation steps, has been well studied in many plants, but not in *S. miltiorrhiza* [22,23,24,25]. This gene directly determines whether anthocyanin can be synthesized and whether plant tissues can be colored. The defects, absence, restriction, and up-regulation of *ANS* can change the color phenotype and the accumulation of anthocyanin, resulting in colorless, white, or other plants phenotypes [22,23,24,25]. Moreover, either the precursor substances (anthocyanin) or catalytic products (leucoanthocyanidin) of *ANS* can act as precursors for producing the subunits of proanthocyanidins. Previous studies have found that the *ANS* gene can determine the accumulation of proanthocyanidins, and affect the production of phenolic acid [26,27,28,29]. In recent years, on the basis of the databases of transcriptome and genome for *S. miltiorrhiza*, more and more genes and transcription factors involved in metabolic regulation have been excavated and studied [1,21,30,31]. Although, some flavonoid structure genes, including *ANS*, involved in flavonoids biosynthesis in *S. miltiorrhiza* have been identified or predicted [32], very little information is available to illuminate the functional characterization of *ANS*. It is extremely important to explore the role *ANS* plays in the color difference between *S. miltiorrhiza* and *S. miltiorrhiza* Bge f. *alba*. The regulatory networks of the secondary metabolism are extremely complex, and an imperceptible change in a small part can affect the whole metabolic network. Therefore, in view of the extremely important roles of *ANS* in plants, it is necessary to survey the functional mechanism of this gene, and its role in the regulation of other non-flavonoids branched metabolic pathways in *S. miltiorrhiza*.

In the present study, *SmANS* in *S. miltiorrhiza* was cloned, and we verified that its expression clearly differed in *S. miltiorrhiza* and *S. miltiorrhiza* Bge f. *alba* during the spatial-temporal development stages. Overexpression of *SmANS* was showed to promote anthocyanin concentration and alter phenolic acids accumulation both in transgenic plantlets of *S. miltiorrhiza* and heterogeneous *S. miltiorrhiza* Bge f. *alba*, respectively, meanwhile it restored the purple-red phenotype of *S. miltiorrhiza* Bge f. *alba* plantlets. These results suggest that *SmANS* is not only a key enzyme gene that controls the purple coloration of the aerial part in *S. miltiorrhiza*, but also acts as a switch that restricts anthocyanin accumulation and purple coloration in *S. miltiorrhiza* Bge f. *alba* white petals and green calyxes, thus providing valuable evidence for the molecular regulatory mechanism of the anthocyanin biosynthetic pathway, as well as the crosstalk between flavonoids and phenolic acid metabolism in *S. miltiorrhiza*.

## 2. Results

### 2.1. Isolation and Bioinformatics Analysis of SmANS

**Figure A1:**
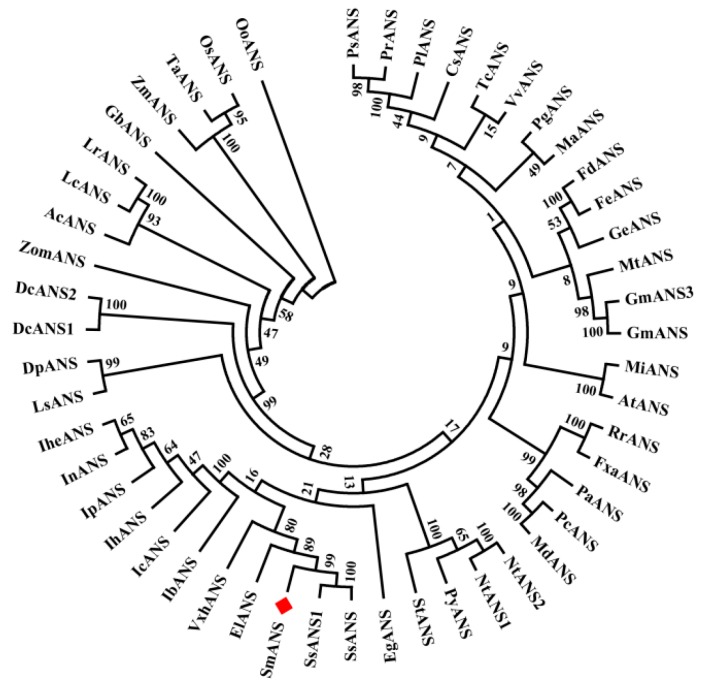
The phylogenetic tree of SmANS protein in *Salvia miltiorrhiza* and ANS protein in other plants. The red marke represents SmANS protein.

Sm*ANS* (GenBank accession number: MK704422) was cloned and identified from purple petals in *S. miltiorrhiza*, it contained a 1110 bp ORF encoding a protein of 369 amino acids with a predicted molecular mass of 41.489 kDa. *SmANS*, a single copy gene in *S. miltiorrhiza*, contained typical ANS-specific conserved motifs and belongs to the 2OG-Fe(II) oxygenase superfamily. In a comparative analysis of the sequences of *SmANS* and *ANS* from *S. miltiorrhiza* Bge f. *alba*, the results indicated that both of them were consistent and had 99.98% identity. To visually describe the evolutionary origins, a phylogenetic tree was constructed using the SmANS and the ANS protein sequences from the other species listed in Table A2. The results showed that SmANS was classified into the eudicots group and clearly differed from the monocotyledonous and phycophyta (*Zostera marina*) groups (Figure A1). The putative amino acid sequence of SmANS had the closest relationship to *Plectranthus scutellarioides* amino acid sequences with 86% identity. It showed 79% identity with the ANS from *Arabidopsis thaliana*.

### 2.2. Spatio-Temporal Expression of SmANS in S. miltiorrhiza and S. miltiorrhiza Bge f. alba

**Figure 2:**
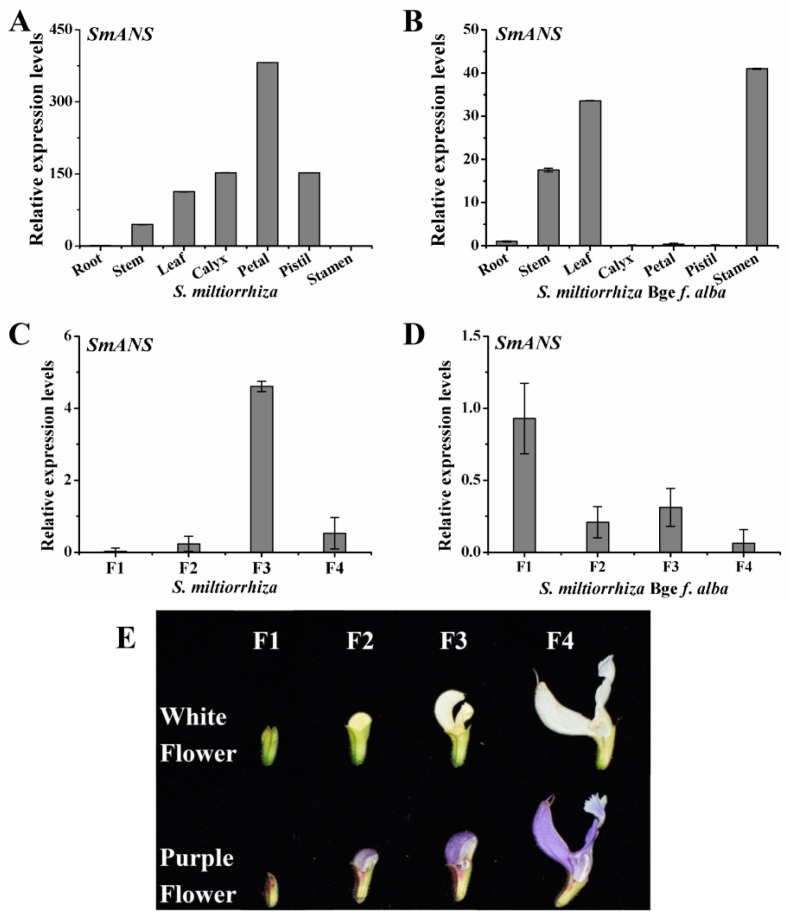
Spatial-temporal expression of *SmANS* in *Salvia miltiorrhiza* and *S. miltiorrhiza* Bge f. *alba*. (**A**) Relative expression levels of *SmANS* in various organs of *S. miltiorrhiza*; (**B**) Relative expression levels of *SmANS* in various organs of *S. miltiorrhiza* Bge f. *alba*; (**C**,**D**) Relative expression levels of *SmANS* in flowers of four flowering stages from *S. miltiorrhiza* and *S. miltiorrhiza* Bge f. *alba*, respectively; (**E**) The purple flowers *S. miltiorrhiza* and white flowers *S. miltiorrhiza* Bge f. *alba* of four flowering stages. F1, young-bud; F2, medium-bud; F3, pre-bloom petal; and F4, full-bloom petal. The vertical bars show the SD values from three independent biological replicates.

Quantitative real-time PCR (qRT-PCR) was conducted to investigate the spatial-temporal expression patterns of *SmANS* in various organs including the root, stem, leaf, calyx, petal, pistil, and stamen, as well as the full flower organs of four flowering stages from two-year-old *S. miltiorrhiza* and *S. miltiorrhiza* Bge f. *alba*. *SmANS* showed remarkably higher expression in the purple petals than the other tissues, and was the lowest in the roots of *S. miltiorrhiza* (Figure 2A). Moreover, *SmANS* was expressed at highest levels in the stamens (with yellow and purple blue pollen) compared with the leaves, stems, and roots, but showed almost no expression in the white petal, green calyx, and pistil of *S. miltiorrhiza* Bge f. *alba* (Figure 2B). During floral development, the expression of *SmANS* in purple flowers of *S. miltiorrhiza* displayed a slowly and then sharply rising trend from the F1 (young-bud) stage to the F3 (pre-bloom petal) stage, whereas it decreased in the F4 (full-bloom petal) stage (Figure 2C). However, the expression levels of *SmANS* in white flowers of *S. miltiorrhiza* Bge f. *alba* exhibited a quickly falling trend from the F1 to F2 (medium-bud) stage, a small increase in the F3 stage (Figure 2D), and a slight decrease again in the F4 stage. These findings uncover that the expression levels of *SmANS* may cause the color phenotypic differences between *S. miltiorrhiza* and *S. miltiorrhiza* Bge f. *alba*.

### 2.3. Subcellular Location of SmANS Protein

**Figure 3:**
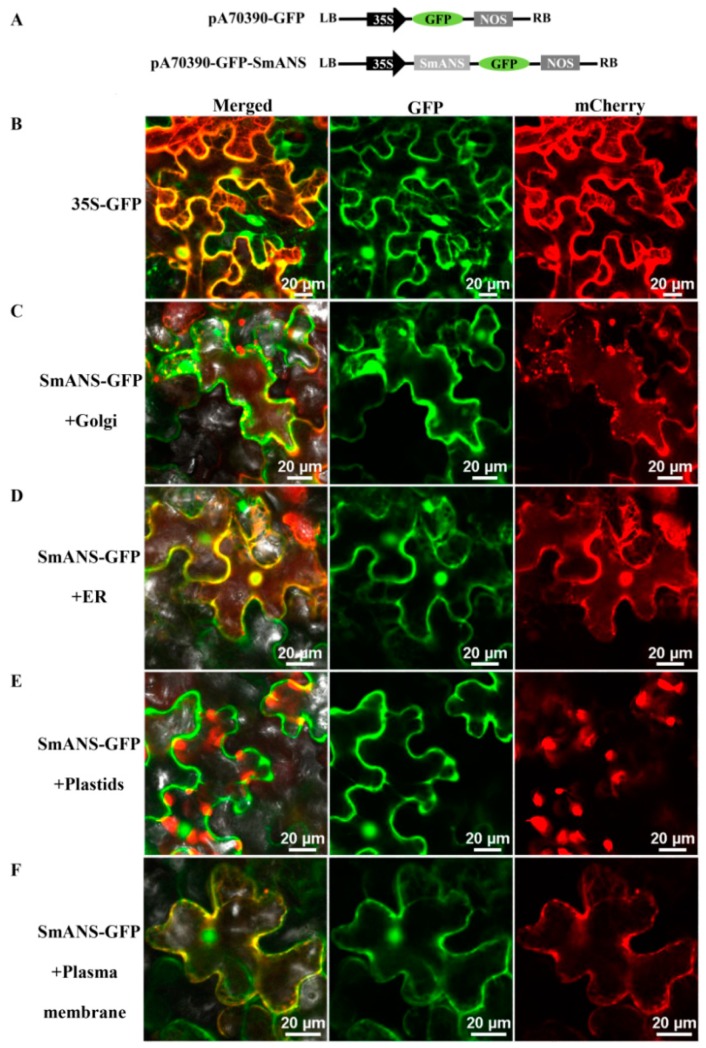
Subcellular localization of SmANS in *Nicotiana benthamiana* leaf epidermal cells. (**A**) Schematic representation of recombinant vector pA70390-GFP-SmANS; (**B**) Subcellular localization of pA70390 with the control (no SmANS inserted); (**C**–**F**) Golgi, endoplasmic reticulum (ER), plastids, and plasma membrane localization of PA70390-GFP-SmANS in *N. benthamiana* leaves epidermal cells (SmANS inserted), respectively. Merged, the overlapping field of GFP and mCherry. GFP, green fluorescent field. mCherry, red fluorescent channel field of Golgi, ER, plastids, and plasma membrane marker. Scale bars, 20 μm.

For localization of the SmANS protein, the ORF of *SmANS* was fused to the 5′-terminus of the GFP reporter gene of pA7 vector controlled by the CaMV 35S promoter. The transient expression system of SmANS-GFP fusion proteins in *Nicotiana benthamiana* was performed to analyze in this study. The GFP fluorescence signals of the pA70390-GFP control were observed throughout the leaf epidermal cells of *N. benthamiana* (Figure 3B). In addition, the GFP fluorescence signals of SmANS were dispersed in the Golgi, ER (endoplasmic reticulum), and plastids of cytoplasm, as well as in the plasma membrane (Figure 3C–F). Moreover, the cell nucleus was full of strong fluorescent signals. Therefore, the SmANS protein was mainly localized in the Golgi, ER, plastids, plasma membrane, and nucleus. These results suggest that the anthocyanins synthesis was catalyzed by a multienzyme complex containing SmANS on the ER membranes, and was finally accumulated in the vacuole through transportation in and out of the cell.

### 2.4. Generation of SmANS-Overexpressing Transgenic S. miltiorrhiza and S. miltiorrhiza Bge f. alba Plantlets

**Figure 4:**
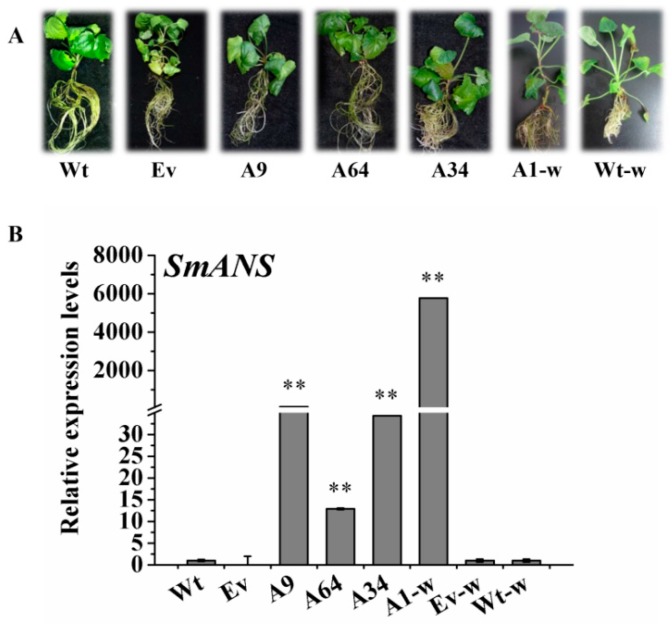
The plantlets and expression levels of *SmANS*-overexpressing in *Salvia miltiorrhiza* and *S. miltiorrhiza* Bge f. *alba* transgenic lines and controls. (**A**) The three-month-old transgenic and controls plantlets of *SmANS*-overexpression in *S. miltiorrhiza* and *S. miltiorrhiza* Bge f. *alba*; (**B**) Relative expression analysis of *SmANS* in the transgenic and control plantlets of *S. miltiorrhiza* and *S. miltiorrhiza* Bge f. *alba*. Wt, the controls that were untransformed wild *S. miltiorrhiza* plants. Ev, the controls that only transformed the empty vector in *S. miltiorrhiza*. A9, A64, and A34, three transgenic lines of *SmANS* -overexpressed in *S. miltiorrhiza*. A1-w, transgenic line of *SmANS*-overexpressing in *S. miltiorrhiza* Bge f. *alba*. Ev-w, the controls that only transformed the empty vector in *S. miltiorrhiza* Bge f. *alba*. Wt-w, the controls that were untransformed wild *S. miltiorrhiza* Bge f. *alba* plants. The vertical bars show the SD values from three independent biological replicates. The ** indicates a very significant differences between transgenic and control plantlets (*p* < 0.01).

In the present study, independent transgenic plantlets exhibiting *SmANS* overexpression in *S. miltiorrhiza* and *S. miltiorrhiza* Bge f. *alba* were obtained and identified using PCR to investigate the potential functional roles of *SmANS* involved in the flavonoids biosynthesis of the two kinds of *S. miltiorrhiza.* The qRT-PCR results demonstrated that the expression levels of *SmANS* were significantly increased not only in overexpressed *S. miltiorrhiza* lines (A9, A34, and A64), but also in heterologously expressed *S. miltiorrhiza* Bge f. *alba* lines (A1-w; Figure 4A). The expression levels of *SmANS* in A9, A34, and A64 plantlets were increased 102.06-, 34.30-, and 12.88-fold compared with the Wt (the controls that were untransformed wild plants of *S. miltiorrhiza*) plantlets, respectively (Figure 4B). However, the *SmANS* expression in the A1-w line was significantly up-regulated up to >5000-fold compared with the Wt-w (the controls that were untransformed wild plants of *S. miltiorrhiza* Bge f. *alba*) plantlets (Figure 4B). Thus, the four efficient overexpressed lines were also selected for further functional analysis.

**Figure 5:**
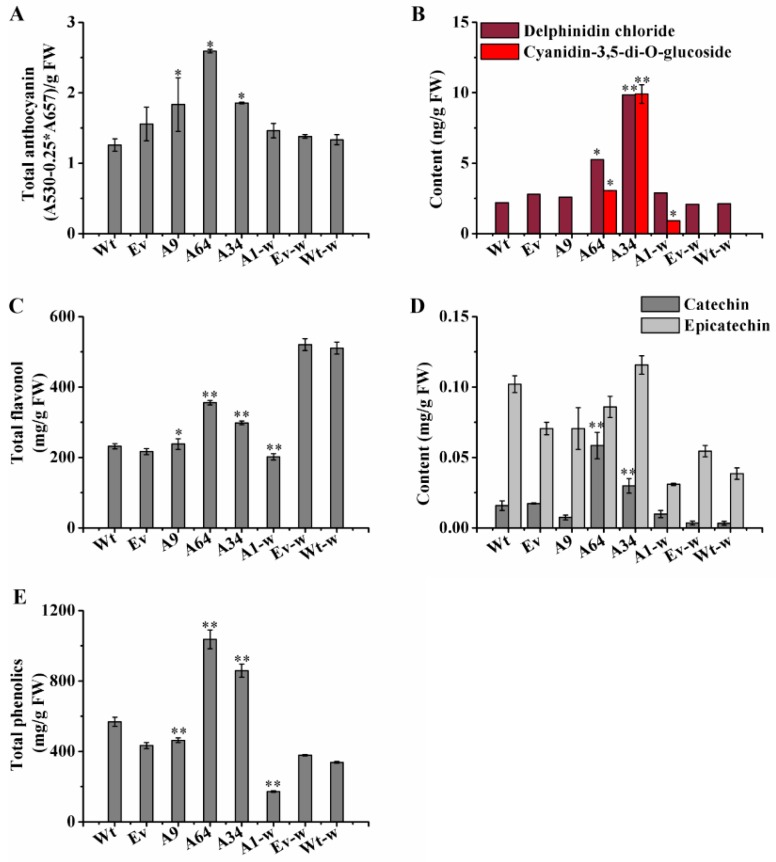
Analysis of flavonoids and total phenolics in transgenic lines. (**A**) The content of total anthocyanin in controls and transgenic lines; (**B**) The contents of delphinidin and cyaniding 3,5-di-*O*-glucoside in controls and transgenic lines; (**C**) The content of total flavonoid from controls and transgenic lines; (**D**) The contents of catechin and epicatechin analyzed by HPLC; (**E**) Total phenolics content from controls and transgenic lines. Wt, the controls that were untransformed wild *S. miltiorrhiza* plants. Ev, the controls that only transformed the empty vector in *S. miltiorrhiza*. A9, A64, and A34, three transgenic lines of *SmANS* -overexpressed in *S. miltiorrhiza*. A1-w, transgenic line of *SmANS*-overexpressing in *S. miltiorrhiza* Bge f. *alba*. Ev-w, the controls that only transformed the empty vector in *S. miltiorrhiza* Bge f. *alba*. Wt-w, the controls that were untransformed wild *S. miltiorrhiza* Bge f. *alba* plants. FW, fresh weight. The vertical bars show the SD values from three independent biological replicates. (*) indicated a significant difference (0.01 < *p* < 0.05). (**) indicated a very significant difference (*p* < 0.01).

Furthermore, compared with the Wt controls, the three transgenic plantlets (A9, A64, and A34) of *S. miltiorrhiza* also showed obvious purple-red coloration, especially on the surface of the leaves. Interestingly, unlike the Wt-w phenotype of *S. miltiorrhiza* Bge f. *alba*, which had completely green leaf margins and stem segments, the A1-w plantlets showed obvious phenotypic changes in color and appearance. The differences in the leaf margins and stem segments in A1-w plantlets included purple-red coloration (Figure 4A), and stronger the stems and leaves as compared to the Wt-w controls. The concentrations of the total anthocyanin were all higher than their respective controls in the four independent transgenic plantlets (Figure 5A). These phenotypic changes indicate that *SmANS* plays extremely important roles in anthocyanin accumulation of *S. miltiorrhiza* and *S. miltiorrhiza* Bge f. *alba*.

### 2.5. SmANS Overexpression Improves the Accumulation of Flavonoids in Transgenic S. miltiorrhiza and S. miltiorrhiza Bge f. alba Plantlets

To determine the effects of *SmANS*-overexpression on the accumulation of main anthocyanin and other flavonoids in transgenic lines, a microplate reader and HPLC were utilized to quantify these contents. Compared with the Wt of *S. miltiorrhiza* or the Wt-w of *S. miltiorrhiza* Bge f. *alba*, the concentration of cyanidin-3,5-di-*O*-glucoside and delphinidin chloride had different increases in the four independent *SmANS*-overexpression lines (Figure 5B). The concentration of cyanidin-3,5-di-*O*-glucoside in A9, Wt, and Vv (controls that only transformed the empty vector in *S. miltiorrhiza*) plantlets was almost undetected. The content of cyanidin-3,5-di-*O*-glucoside significantly increased, reaching 3.93- and 10.78-fold in the A64 and A34 lines, respectively, of Wt content. Meanwhile, compared with the Wt plantlets, the content of delphinidin chloride was remarkably improved up to 0.39-fold (A9), 3.06-fold (A64), and 7.66-fold (A34) of the Wt content, respectively. Moreover, the accumulation of cyanidin-3,5-di-*O*-glucoside and delphinidin chloride was respectively increased by 3.01 ng/g FW and 0.78 ng/g FW in A1-w line as compared to the Wt-w content. These results indicate that *SmANS* was the key gene controlling anthocyanin biosynthesis in the *S. miltiorrhiza* plantlets.

*SmANS* overexpression also enhanced the accumulation of total flavonoid in the three transgenic *S. miltiorrhiza* plantlets (A9, 64, and 34), reaching 1.03-, 1.53-, and 1.29-fold, respectively, of the Wt content. The total phenolics content increased only in *S. miltiorrhiza* transgenic plantlets (A64 and A34), up to 1.82-fold and 1.50-fold, respectively, of the Wt content (Figure 5E). However, the total flavonoid and total phenolics contents in the A1-w lines were reduced by 60.45%, and 48.92% of the Wt-w content, respectively (Figure 5C,E). In addition, the contents of catechin in the proanthocyanidin biosynthetic pathway were increased in part transgenic lines such as A64, A34, and A1-w, which had high anthocyanidin accumulation (Figure 5D). However, the epicatechin content was reduced in all lines except A34 (Figure 5D). These findings revealed that *SmANS*-overexpression could improve the accumulation of the other flavonoids such as flavonols and proanthocyanidin monomers in parts of the transgenic lines, and point to the metabolic flux flooding into the flavonoids branched pathway.

### 2.6. Overexpression of SmANS Up-Regulates Parts of Pathway Genes Involved in Flavonoids Biosynthesis

**Figure 6:**
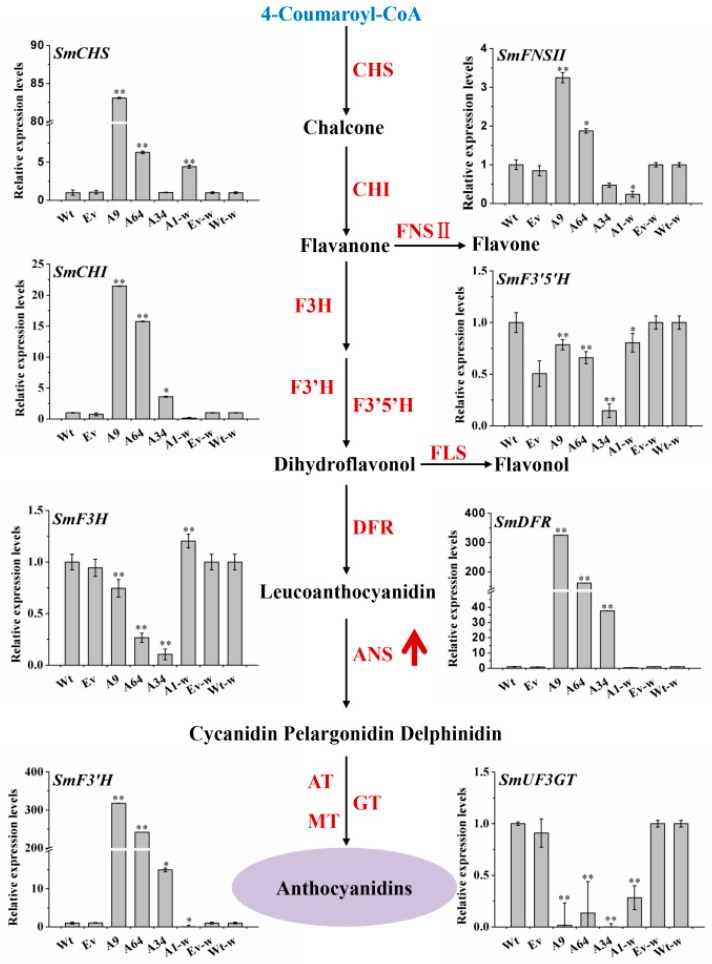
The expression pattern of the flavonoids structure genes in the transgenic lines. The black solid arrows represent the catalytic reactions. The upward red arrow represents the overexpression of the *SmANS* gene. Wt, the controls that were untransformed wild *Salvia miltiorrhiza* plants. Ev, the controls that only transformed the empty vector in *S. miltiorrhiza*. A9, A64, and A34, three transgenic lines of *SmANS*-overexpressed in *S. miltiorrhiza*. A1-w, transgenic line of *SmANS*-overexpressing in *S. miltiorrhiza* Bge f. *alba*. Ev-w, the controls that only transformed the empty vector in *S. miltiorrhiza* Bge f. *alba*. Wt-w, the controls that were untransformed wild *S. miltiorrhiza* Bge f. *alba* plants. The vertical bars show the SD values from three independent biological replicates. (*) indicated a significant difference (0.01 < *p* < 0.05). (**) indicated a very significant difference (*p* < 0.01).

The flavonoids-branched pathway is derived from the phenylpropanoid biosynthetic pathway. To further confirm the effects of *SmANS*-overexpression on the flavonoids pathway, we analyzed the transcriptional levels of the 11 genes involved in the flavonoids pathway in the transgenic *S. miltiorrhiza* and *S. miltiorrhiza* Bge f. *alba* plantlets. The results are shown in Figure 6. The overexpression of *SmANS* significantly decreased the relative expression levels of the phenylpropanoid pathway genes (*SmPAL*, *SmC4H*, and *Sm4CL*) in all of the transgenic *S. miltiorrhiza* and *S. miltiorrhiza* Bge f. *alba* plantlets. However, the expression levels of *SmCHS*, *SmCHI*, *SmF3’H*, and *SmDFR* involved in the flavonoids pathway significantly increased in the three overexpressing *S. miltiorrhiza* plantlets as compared with the Wt expression levels. The expression levels of *SmF3H*, *SmF3′5′H*, and *SmUF3GT* were down-regulated in the *S. miltiorrhiza* plantlets as compared with the Wt group. In addition, the expression levels of *SmFNSII* (flavonols synthase) were increased in the A9 and 64 lines but reduced in the A34 lines as compared to the Wt lines. In addition, the heterologous overexpression of *SmANS* in *S. miltiorrhiza* Bge f. *alba* distinctly improved the expression levels of *SmCHS* and *SmF3H*. Both the accumulation of total flavonols and the expression levels of *SmFNSII* were reduced in the A1-w line as compared to the Wt-w controls. These results indicate that *SmANS*-overexpression improved the transcriptional levels of most pathway genes in flavonoids biosynthesis, and down-regulated the pathway genes in phenylpropanoid biosynthesis in transgenic lines.

### 2.7. SmANS Overexpression Indirectly Affects the Salvianolic Acids Branched Pathway

**Figure 7:**
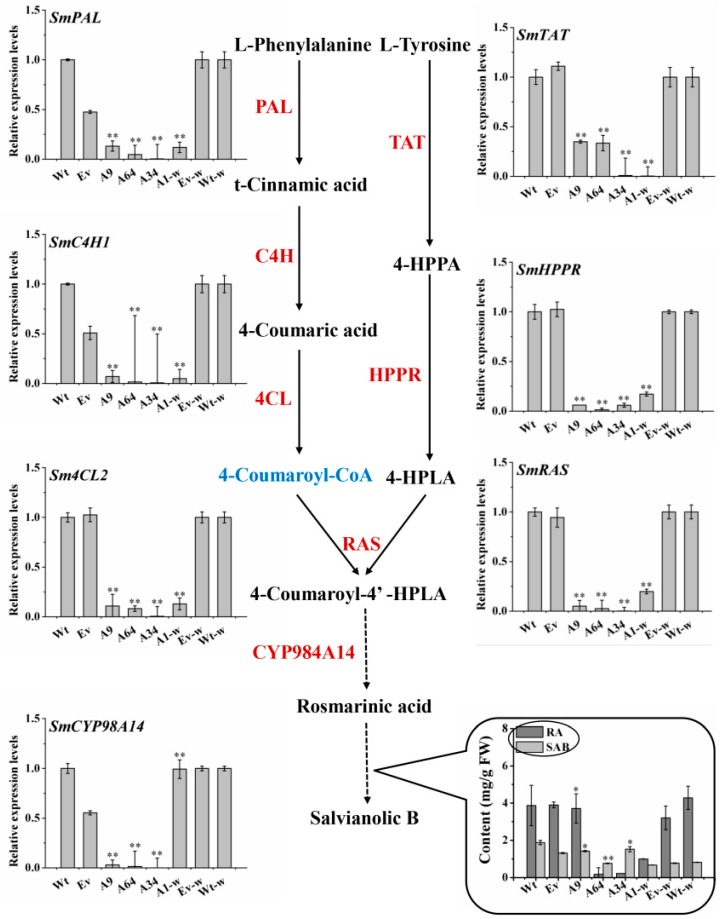
The contents of phenolic acids and expression pattern of the structure genes involved in salvianolic acid biosynthesis in transgenic lines. Solid arrows and dashed arrows represent single-step reactions and multiple steps reactions, respectively. Wt, the controls that were untransformed wild *S. miltiorrhiza* plants. Ev, the controls that only transformed the empty vector in *S. miltiorrhiza*. A9, A64, and A34, three transgenic lines of *SmANS*-overexpressed in *S. miltiorrhiza*. A1-w, transgenic line of *SmANS*-overexpressing in *S. miltiorrhiza* Bge f. *alba*. Ev-w, the controls that only transformed the empty vector in *S. miltiorrhiza* Bge f. *alba*. Wt-w, the controls that were untransformed wild *S. miltiorrhiza* Bge f. *alba* plants. The vertical bars show the SD values from three independent biological replicates. (*) indicated a significant difference (0.01 < *p* < 0.05). (**) indicated a very significant difference (*p* < 0.01).

To explore the changes in salvianolic acids pathway in the *SmANS*-overexpression plantlets, the contents of SAB and RA, as well as the transcriptional levels of the key genes involved in the salvianolic acids branched pathway were analyzed. HPLC results showed that the accumulation of RA and SAB in the three-month-old overexpressed *S. miltiorrhiza* and *S. miltiorrhiza* Bge f. *alba* plantlets were reduced in a different manner as compared to the controls (Figure 7). The transcriptional levels of *SmPAL*, *SmC4H1*, *Sm4CL2*, *SmTAT*, *SmHPPR*, *SmRAS*, and *SmCYP98A14* involved in the phenylpropanoid and tyrosine-derived branches were significantly decreased in the *SmANS* overexpressed lines (A9, A64, A34, and A1-w) as compared to the Wt or Wt-w lines, respectively (Figure 7). The expression pattern of the eight genes involved in SAB biosynthesis was consistent with the accumulation of RA and SAB. The results suggest that the SAB branched pathway was indirectly affected by *SmANS* in the flavonoids pathway.

### 2.8. SmANS-Overexpression Down-Regulates the Expression Levels of Part Transcriptional Regulation Factors

**Figure 8:**
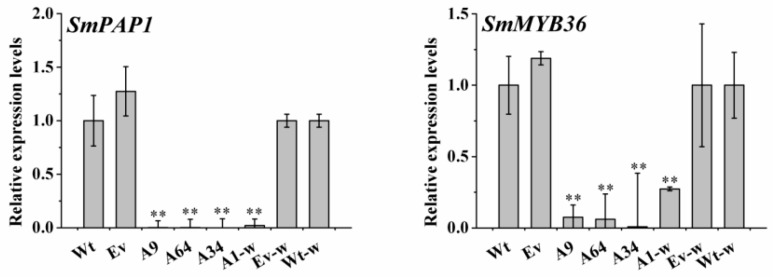
The expression levels of transcription factors in transgenic lines. Wt, the controls that were untransformed wild *S. miltiorrhiza* plants. Ev, the controls that only transformed the empty vector in *S. miltiorrhiza*. A9, A64, and A34, three transgenic lines of *SmANS* -overexpressed in *S. miltiorrhiza*. A1-w, transgenic line of *SmANS*-overexpressing in *S. miltiorrhiza* Bge f. *alba*. Ev-w, the controls that only transformed the empty vector in *S. miltiorrhiza* Bge f. *alba*. Wt-w, the controls that were untransformed wild *S. miltiorrhiza* Bge f. *alba* plants. The vertical bars show the SD values from three independent biological replicates. (**) indicated a very significant difference (*p* < 0.01).

The enzymatic activity of *ANS* and other genes involved in flavonoids was regulated by transcriptional regulation factors including MYB, bHLH, and WD. In the present study, the expression of *SmPAP1* and *SmMYB36* were all markedly decreased in the four *SmANS*-overexpression lines, as compared with the wild controls of *S. miltiorrhiza* and *S. miltiorrhiza* Bge f. *alba*, respectively (Figure 8). These suggested that the molecular and metabolic flow between the flavonoid pathway and salvianolic acids pathway was regulated by the two regulation factors.

## 3. Discussion

Anthocyanin and other flavonoids are the main pigments that determine the coloration of numerous plant tissues and organs. The key enzyme, *ANS* is responsible for the formation of anthocyanin from leucoanthocyanidin in the flavonoids biosynthetic pathway, and has been well studied in most plants [33,34,35,36]. Although, *SmANS* has been cloned and analyzed to its tissue-specific expression in *S. miltiorrhiza* [32], the functional molecular characteristics of this key gene are not well known. In *S. miltiorrhiza*, flavonoids and phenolic acids are all produced via the biosynthetic pathway of phenylpropane. In addition, *S. miltiorrhiza* is a model plant for traditional Chinese medicine plant research in view of its significant medicinal value, thus, we need to focus on the controlling influence of *SmANS* overexpression on the phenylpropanoid pathway, particularly relative to the accumulation of flavonoids and phenolic acids to survey the crosstalk of the flavonoids and phenolic acids pathways.

In this study, *SmANS* was successfully cloned and characterized from the purple flowers in *S. miltiorrhiza*. Using a comparative analysis of the expression pattern of *SmANS* in various organs of the *S. miltiorrhiza* and *S. miltiorrhiza* Bge f. *alba* during the full-bloom stage, we found that *SmANS* was highly expressed especially in the purple petals of *S. miltiorrhiza*, whereas, it showed almost no expression in the white petals, green calyxes, or pistils in *S. miltiorrhiza* Bge f. *alba*, suggesting that *SmANS* could be responsible for the accumulation of anthocyanin and the purple coloration. The coloration of flower and other organs is controlled or determined by the *ANS* transcriptional levels, which has been illuminated and verified in other plants. For instance, in *Solanum melongena* L, *FAS* is expressed more strongly in purple flowers than in white flowers, and controls the flower color [37]. In carrot taproots, *LDOX1*/*LDOX2* along with other anthocyanin genes, is highly expressed in purple cultivars but not expressed or barely in non-purple cultivars [38]. Similar profiles in tree peony leaf, where *ANS* is the key gene that controls the anthocyanin levels, were more highly expressed in purplish red leaves than in yellowish green leaves [39]. Additionally, in Asiatic hybrid lilies, *ANS*, *CHS*, and *DFR* were expressed more highly in pigmented parts than in non-pigmented parts [40]. These suggested that *SmANS* may be a key gene in determining purple coloration degree in *S. miltiorrhiza*, also may be a limiting factor in the formation of the non- purple phenotype in *S. miltiorrhiza* Bge f. *alba*.

The accumulation of dominant anthocyanin occurs in the vacuole, but the synthesis of anthocyanin takes place on the cytosolic surface of the ER [41]. Transient expression analyses using tobacco leaf epidermal cells have shown that SmANS protein fused to GFP is not only localized in the cytosolic organelles (e.g., ER, plastids, and Golgi), but also in the plasma membrane and cell nucleus. This multi-subcellular-localization of ANS protein is also found in grape berries [42]. Much evidence also has proved that flavonoids structural genes containing *ANS* synthesized anthocyanin on the ER membranes by flavonoids biosynthetic enzyme complexes [43,44]. The SmANS protein distributed in different subcellular organelles suggest that the anthocyanin and other flavonoids may be synthesized in the ER, and stored in the vacuole using some kinds of transporter to move within and between cells.

To examine the possible functional roles of *ANS* in flavonoids biosynthesis in *S. miltiorrhiza*, we obtained transgenic plantlets both *S. miltiorrhiza* and *S. miltiorrhiza* bge f. *alba* with *SmANS* overexpression. By comparing the transgenic plantlets with their controls, respectively, we found that *SmANS* overexpression resulted in purple-red color phenotype (Figure 4B). It was closely linked with increases in cyanidin-3,5-di-*O*-glucoside, delphinidin, and total anthocyanin in the transgenic plantlets of two types of *S. miltiorrhiza* (Figure 5). The cyanidin-3,5-di-*O*-glucoside existed in the three-month-old plantlets of the A64, A34, and A1-w lines but not in wild-type or empty vector controls, and its concentration was positive relative to the expression levels of *SmF3’H*, which determines the cyaniding-based anthocyanin biosynthesis. The cyanidin-based anthocyanin was activated by the *SmANS* overexpression. In addition, the biosynthesis of delphinidin-based anthocyanin was enhanced by the *SmANS* overexpression. The purple-red phenotype in our transgenic plantlets was determined by the summation of the cyaniding-derivative and delphinidin-derivative. Previous studies have also shown that when *ANS* is overexpressed or up-regulated, carbon flux is directed towards anthocyanin biosynthesis [26,29,36,45,46]. The *ANS* enzyme gene has been shown to act as a key switch on the synthesis of anthocyanin in many other plants, such as red-fleshed kiwifruit [23], wintersweet flower [35], and crabapple petals [47]. Moreover, heterologous overexpression of *SmANS* in *S. miltiorrhiza* Bge f. *alba* restored color phenotype in leaf edges, and stem internodes followed the increase in anthocyanin (Figure 4B). Hence, we further confirmed that the expression of *SmANS* was restricted in three-month-old wild plantlets of *S. miltiorrhiza* Bge f. *alba*. Since *SmANS* expression levels were also limited in the white flowers and green calyxes of *S. miltiorrhiza* Bge f. *alba*, we propose that *SmANS* is a switch on anthocyanin biosynthesis in *S. miltiorrhiza* and *S. miltiorrhiza* Bge f. *alba* plantlets.

The metabolic crosstalk of the flavonoids with other pathways acts to mediate plants adaptation to their real estate [48]. The up- or down-regulation of the gene involved in all these pathways would reallocate the metabolic flux to each branched pathway, according to the source-sink theory. Phenolic acids branched pathway in *S. miltiorrhiza* are thought to be produced through two parallel pathways: The phenylpropanoid-derived and tyrosine-derived pathways. SAB biosynthesis is derived from its RA precursor. Although the accumulation of anthocyanins was enhanced, the accumulation of RA and SAB in *SmANS*-overexpressed plantlets was decreased (Figure 7). The expression of the key genes (*SmPAL*, *SmC4H1*, *Sm4CL2*, *SmTAT*, *SmHPPR*, *SmRAS*, and *SmCYP98A14*) involved in SAB biosynthesis was down-regulated, which was positive relative to the phenolic acids concentration (Figure 7). However, *SmPAL*, *SmC4H1*, and *Sm4CL2* were also key genes in the early steps of flavonoids biosynthesis, as in SAB biosynthesis. These findings indicate that the enormous metabolic flux directly to the flavonoids-branched pathway and especially to the anthocyanin sub-branch, resulted in insufficient common source storage for phenolic acids biosynthesis. The metabolic flux between the flavonoids-branched pathway and SAB pathway could explain the effect of *SmCHS* silencing in *S. miltiorrhiza* [13]. Both the *SmCHS* port gene and its downstream genes, such as *SmANS*, have similar effects on the non-flavonoids branched pathway by virtue of regulating the metabolic flow distribution.

The similarity was that the metabolic crosstalk existed in both the anthocyanin and proanthocyanidins branched pathways because they share common substrates: Anthocyanin or leucoanthocyanidin. The transgenic plantlets with *SmANS*-overexpression had differing influence on the accumulation of the primary monomers (catechin and epicatechin) in proanthocyanidins biosynthesis, which was associated with the anthocyanin levels. Catechin was elevated in the A34, A64, and A1-W lines, which produced sufficient anthocyanin. However, the epicatechin content was improved only in the A34 line, which accumulated the highest levels of cyanidin-3,5-di-*O*-glucoside and delphinidin chloride. A sufficient anthocyanin intermediate could satisfy epicatechin monomers in proanthocyanidins biosynthesis. Owing to *SmANS* overexpression, the anthocyanin intermediate first met the supply of anthocyanidin synthesis, and then contributed additional parts to the epicatechin monomer biosynthesis. Here, the metabolic flow may have a preference for the anthocyanin biosynthesis.

The key genes of the secondary metabolic pathway are thought to be regulated by the relative transcription factors through interacting with *cis*-elements of these genes. *SmPAP1* and *SmMYB36* in *S. miltiorrhiza* have been reported to participate in regulating phenolic acids biosynthesis, yet the latter belonging to the S6 AtMYBs may be involved in anthocyanin biosynthesis [2,21]. In the four independent *SmANS*-overexpression lines, the transcription levels of *SmPAP1* and *SmMYB36* were significantly down-regulated, relative to the controls (Figure 8). It has been reported that *SmPAP1* activates the promoters of *SmPAL1* and *SmC4H*, and overexpression of this transcription factor can induce phenolic acids biosynthesis and accumulation in transgenic *S. miltiorrhiza* Bge f. *alba* roots [2]. The down-regulation of *SmPAP1* in *SmANS*-overexpression lines was positively related with the decrease of RA and SAB concentrations as well as the expression of the key genes involved in SAB biosynthesis (Figure 7 and Figure 8). Our analysis further verified that *SmPAP1* positively regulated phenolic acids biosynthesis and accumulation *in S. miltiorrhiza* plantlets. Previous research in our laboratory confirm that *SmMYB36*-overexpression inhibits phenolic acid and flavonoid biosynthesis in *S. miltiorrhiza* hairy roots [20]. It appears that *SmMYB36* might be a negative regulator in phenolic acid biosynthesis. Our findings showed that *SmANS* overexpression decreased the expression of key genes (including *SmPAL*, *SmC4H*, *Sm4CL*, *SmRAS*, and *SmCYP98A14*) in phenolic acid biosynthesis and the accumulation of SAB and RA, whereas it increased the expression of most flavonoids genes and the concentrations of cyanidin-3,5-di-*O*-glucoside and delphinidin chloride. In addition, the transcriptional levels of *SmMYB36* showed a negative correlation with the anthocyanidin concentration and total flavone in our transgenic lines, which indicated that this transcriptional factor was negatively regulated in anthocyanin and other flavonoids biosynthesis in *S. miltiorrhiza*. Thus, we propose that the expression levels of *ANS* are regulated by *SmMYB36*. This regulation may interact with other transcription factors (bHLH and WD) to form MBW complexes, which regulate anthocyanin biosynthesis [17,18,49,50,51]. The results of the present study imply that the *SmMYB36* participates in regulating the metabolism flow between anthocyanin and SAB biosynthesis in the *SmANS*-overexpressed lines.

In conclusion, the analysis of spatio-temporal expression of *SmANS* suggested that this gene was one of the key genes in the purple formation in *S. miltiorrhiza*, and its lower or limited expression might be the most critical reason for the formation of the white petals and green calyx in *S. miltiorrhiza* Bge f. *alba*. Then we have characterized the function of *SmANS* by overexpression in *S. miltiorrhiza* and *S. miltiorrhiza* Bge f. *alba*. Overexpression of *SmANS* inhibited phenolic acid biosynthesis, enhanced the anthocyanin accumulation, and even improved flavonols and proanthocyanidins biosynthesis in parts plantlets, with the result that the metabolic flux was directed to the flavonoids pathway, and thereby reduced in phenolic acids biosynthesis. The purple-red phenotype in *SmANS*-overexpressed lines and the tissue-specific expression differences indicated that the *SmANS* was a “switch” on anthocyanin biosynthesis and color formation in *S. miltiorrhiza* and *S. miltiorrhiza* Bge f. *alba*. These results offer a promising strategy for producing beneficial flavonoids. The findings of this study aid better understanding of the roles of *SmANS* on color formation and the crosstalk of the flavonoids and phenolic acids biosynthesis pathways. Further experiments are needed to verify the transcription factors interacting with *SmANS*. This study provides new evidence for the molecular regulation mechanism involved in flavonoids and phenolic acids biosynthesis.

## 4. Materials and Methods 

### 4.1. Plant Materials

Various fresh tissues of two-year-old *S. miltiorrhiza* and *S. miltiorrhiza* Bge f. *alba* were collected in May during flourishing florescence from the Institute of Soil and Water Conservation (Yangling, China). Species verification was performed by Zongsuo Liang of Northwest A&F University. Roots, stems, leaves, calyxes, petals, pistils, and stamens tissues were used for the analysis of the tissue-specific expression pattern of *SmANS*. In addition, the full flowers during the four flowering stages (F1: young-bud, F2: medium-bud, F3: pre-bloom petal, and F4: full-bloom petal) from the two types of *S. miltiorrhiza* were selected to investigate the temporal expression pattern of *SmANS*. 

The mature seeds of *S. miltiorrhiza* and *S. miltiorrhiza* Bge f. *alba* cultivars were provided by Shaanxi Tasly plant medicine Co., Ltd. (Shangluo, China), and used to obtain sterile plantlets in the MS medium (pH 5.8) supplemented with 3% sucrose and 0.7% agar as previously described [3,52]. Then these sterile plantlets were sub-cultured in 1/2 MS medium (pH 5.8) supplemented with 3% sucrose and 0.75% agar. Leaves of the plantlets sub-cultured for 30 days were finally provided to the genetic transformation of *S. miltiorrhiza* and *S. miltiorrhiza* Bge f. *alba* cultivars through the *A. tumefaciens*-mediated method.

### 4.2. Total RNA and DNA Extraction 

Total complete RNA was isolated from various tissues of *S. miltiorrhiza* and *S. miltiorrhiza* Bge f. *alba* by using the RNAprep pure Plant Kit (TIANGEN, China), and then reversely transcribed to generate cDNA according to the instruction of PrimeScript^TM^ RT Reagent Kit (Takara, Japan). The genomic DNA from the two kinds of *S. miltiorrhiza* and all the transgenic lines was isolated by employing the Genomic DNA Isolation Kit (Cowin Biotech, Beijing, China). The quality and concentration of the RNA and DNA were measured by means of gel electrophoresis and the nucleic acid spectrometer (NanoDrop ND-1000, Thermo Scientific, Woburn, MA, USA).

### 4.3. Isolation and Bioinformatics Analysis of SmANS

To obtain homologous fragments of *SmANS* from purple flowers in *S. miltiorrhiza*, the degenerate primers for Half’s Nest-PCR amplification were designed based on the conserved domains of ANS protein of other plant species using CODEHOP primer designer [53]. The first round amplification introduced the cDNA templates of purple flowers with the primers *ANS*-F1 and *ANS*-R2, and then the products were used as the templates for the second round PCR amplification with the primers *ANS*-F1 and *ANS*-R3. Based on the obtained fragments sequences and *S. miltiorrhiza* transcriptome database [30], we designed one pair of specific primers (*SmANS*-F1 and *SmANS*-R2) to isolate the length cDNA of *SmANS* from the purple flowers of *S. miltiorrhiza*. The primers in this study are all shown in Table A1. The ORF of *ANS* from *S. miltiorrhiza* was found with ORF (open reading frame)-finder (https://www.ncbi.nlm.nih.gov/orffinder/). Amino acid sequences of *SmANS* were performed in the DNAStar 7.1. The MEGA v 5.10 software was performed for the construction of phylogenetic trees by employing the neighbor-joining method with 1000 bootstrap replicates. The species in the phylogenetic trees are listed in Table A2. 

### 4.4. QRT-PCR Analysis

QRT-PCR was performed on a CFX96 Real-time PCR system (Bio-RAD, Hercules, CA, USA) using the SYBR Premix Ex TaqTM II Kit (TaKaRa, Shiga, China), with *SmActin* as an internal reference to normalize the control samples [54]. All experiments were performed in triplicate, and the relative expression levels of the genes were calculated using the 2^−ΔΔ*C*t^ method [55]. The gene-specific primers of the genes involved in flavonoids biosynthesis are shown in Table A1. The other primers of the phenylpropanoid biosynthesis pathway genes (*SmPAL*, *SmC4H*, *Sm4CL*, *SmTAT*, *SmHPPR*, *SmRAS*, *SmCYP98A14*, *SmCHS*, *SmF3’5’H*, and *SmPAP1*) were used according to previous reports [2,3,13].

### 4.5. Subcellular Localization Analysis

In this study, the complete ORF sequence of *SmANS* containing the *Sal*I and *Spe*I restriction enzymes sites was firstly amplified with gene-specific primers *SmANS*-GFPf and *SmANS*-GFPr, and then sub-cloned into the PMD 19T-Simple vector. The PCR product of *SmANS* was fused to the *Sal*I-*Spe*I restrictions sites of the pA7-GFP with the CaMV 35S promoter and *GFP* gene. Both the pA7-GFP-SmANS and pCAMBIA0390 plasmid were digested by *Hind*III-*Eco R*I restriction enzymes and then linked to produce the recombinant plasmid p0390A7-GFP-SmANS. The identified positive recombinant plasmid, markers (Golgi: pG-rk-CD3-967, ER: pER-rb-CD3-960, plastids: pt-rk-CD3-999, plasma membrane: pm-rk CD3-1007) [56], and pA7-0390 empty plasmid were finally transformed into *A. tumefaciens* strain EHA105, respectively, and further expand-cultured in the dark to an OD_600_ of 0.6 at 28 °C. Then the precipitate was collected and re-suspended in injection solution (0.5 M MES/KOH pH 5.6, 100 mM MgCl_2_, 100 mM acetosyringone) to an OD_600_ of 1.0, and kept in the dark for 3 h [57]. Finally, the suspensions of pA7-GFP-*SmANS*-0390 and a different marker were mixed in equal ratios, respectively, and injected into the leaves of a 30-day-old *N. benthamiana*. The empty pA7-GFP-0390 plasmids were used as a control. After four days of incubation, the leaves of *N. benthamiana* were observed under a confocal laser scanning microscope (Nikon A1R, Tokyo, Japan). The relative GFP fusions signals were excited at wavelengths of 488 nm and 561 nm, respectively. 

### 4.6. Acquisition of Positive Transgenic Lines

To construct the plant-overexpressing vector, the complete ORF of *SmANS* was amplified with gene-specific primers (Table A1), and then cloned into the restriction sites *BamH*I and *Kpn*I of the pCAMBIA2300 binary vector. 

The Agrobacterium-mediated transformation method was performed for transgenic *S. miltiorrhiza* and *S. miltiorrhiza* Bge f. *alba* plantlets according to previously described methods [3,52], with minor modifications. The identified recombinant plasmid *SmANS*-2300 and pCAMBIA2300 empty vector were transformed into an *A. tumefaciens* strain (EH105), respectively. Three-month-old plantlets were prepared for PCR identification, PCR screening, expression analysis, and quantification of the secondary metabolites of flavonoids, phenolic acids, and PAs. The primers used for the PCR identification of transgenic lines are listed in Table A1.

### 4.7. Extraction and Determination Anthocyanin, Catechin, and Epicatechin 

A liquid nitrogen grinding sample (0.2 g) was extracted in 1 mL of acidified methanol (1% HCl, *v*/*v*) at 4 °C in the dark for 12 h with manual shaking three times. The extract liquor was centrifuged at 1000 rpm at 4 °C for 2 min, the supernatant was collected, and the precipitate was re-extracted in an equal volume of extracting solution. Each extract was performed in triplicate. The combined-supernatant solutions were evaporated to concentrate using a rotary evaporator (RE52AA, Yarong, Shanghai, China) at 30 °C. Then the concentrate was re-dissolved in 0.8 mL extracting solution and filtered through a 0.22 μm microporous membrane (Jinteng, Tianjin, China) for the following analysis. 

Quantification of the total anthocyanin was performed according to previously reported protocols [2,19,58], with minor modification. Absorption of the extracts at wavelengths of 530 nm and 657 nm was measured at 25 °C using Multimode Microplate Readers (Spectra Max M 2, Hercules, CA, USA). The concentration of the total anthocyanin was calculated according to the following formula: (A_530_ − 0.25 × A_657_)/FW. A_530_ and A_657_ are the absorptions at the 530 nm and 657 nm wavelengths, respectively. FW is the fresh weight (in grams) of the plant tissues used for the extraction.

The contents of delphinidin chloride, cyanidin-3,5-di-*O*-glucoside, catechin, and epicatechin were all detected on Waters HPLC 1525 system (Milford, MA, USA), equipped with a Waters 2996 photodiode array detector. Chromatographic separation was performed with a Waters Xbridge C18 column (4.6 mm × 250 mm, 5 μm particle size) a column temperature of 30 °C and 20 μL injection volume at a flow rate of 1.0 mL/min. The mobile phase consisted of 0.5% trifluoroacetic acid in water (A) and 100% acetonitrile (B). The gradient profile as follows (all concentrations are *v*/*v*): 0–3 min, 0% to 86% A; 3–10 min, 86% to 85% A; 10–30 min, 85% to 70% A; 30–45 min, 70% to 95% A; 45–46 min, 95% to 100% A. The spectra were scanned from 210 nm to 600 nm. The detection of anthocyanin, catechin, and epicatechin was carried out at 520 nm, 280 nm and 280 nm, respectively. All reagents were of HPLC grade. Standards of delphinidin chloride and cyanidin-3,5-di-*O*-glucoside were purchased from Sigma-Aldrich (St. Louis, MO, USA). Standards catechin and epicatechin were purchased from Yuanye Shanghai.

### 4.8. Extraction and Determination of Phenolic Acids and Total Flavonoid

To quantity the main phenolic acid contents RA and SAB in the transgenic and controls, HPLC was performed according to the general method in our laboratory [59]. The only difference is that 0.2 g sample obtained through the liquid nitrogen grounding method, was extracted in 4 mL 70% methanol for 12 h, and filtered through a 0.22 μm microporous membrane (Jinteng, Tianjin, China) for the RA and SAB analysis. Part of the extract was also used for the measure of total phenolics and total flavonoid according to the methods of previously reported [13,31], using the standard gallic acid (Sigma, St. Louis, MO, USA) and quercetin (Sigma, St. Louis, MO, USA), respectively.

### 4.9. Statistical Analysis

The statistical analyses of qRT-PCR and secondary metabolic components detection data were performed by SPSS (version 16.0). *p* < 0.05 was considered statistically significant.

## Appendix A

**Table A1:** The primers used in this study.

Primer	Sequence (5′ to 3′)	Note
*ANS*-F1	CGTGATGCACCTGGTCaaycayggnrt	Homologous cloning of *SmANS*
*ANS*-R2	GGACAGGATCTCGATGGTGtcnccdatrtg	
*ANS*-R3	CAGGCCGGGCaccatrttrtg	
*SmANS*-F1	CTGAAGCGGGAAGGGACT	Full length cDNA cloning of *SmANS*
*SmANS*-R1	GAGGACCTAACAGAACTCGCC	
*SmANS*-GFPf	TTTTTTGTCGACATGGTTGCTACACCGG (*Sal*I site underlined)	Construction of subcellular localization vector
*SmANS-GFP*r	GGGACTAGTGCACTCGATTTATCATAATCAGGC (*Spe*I site underlined)	
*SmANS*-OE-F	TTTTGGATCCATGGTTGCTACACCGG (*BamH*I site underlined)	Construction of overexpression vector
*SmANS*-OE-R	CGCGGGGTACCTCAACTCGATTTATCA (*Kpn*I site underlined)	
2300*NPTII*-F	ATACCGTAAAGCACGAGGAAG	Transgenic lines identification
2300*NPTII*-R	CTGAAGCGGGAAGGGACT	
CaMV35S-F	GAGGACCTAACAGAACTCGCC	
*SmANS*-O-R	CCAAATAGACAAGTCCCTCT	
*SmCHI*-F	TTCCATTCCAGAGCAAGGCG	qRT-PCR analysis
*SmCHI*-R	CCTTGCAGTAGGCGACACTCCTT	
*SmUF3GT*-F	AGCCAAAACCGCCCTAATTCAGTA	
*SmUF3GT*-R	ATGGGAATCCGCATGTTTCTAGG	
*SmFSNII*-F	TCGACCCGATCATCACCAAAGAC	
*SmFSNII*-R	GGGAATCCGCATGTTTCTAGG	
*SmDFR*-F	TAAACTTCATCAGCATCATACCACC	
*SmDFR*-R	TCCTTGCTTTATGATGGAGTAGTG	
*SmANS*-F	CTTGTCTATTTGGCCCAAGCACC	
*SmANS*-R	CTGAGGGCATTTCGGGTAGAAG	
*SmF3H*-F	AAAGCGTGCGTTGATATGG	
*SmF3H*-R	GATCCATGTATTACCACCATCC	
*SmF3’H*-F	TGCCACGAACGCAATAGCTC	
*SmF3’H*-R	TTGAATACTCCAGCCAACGACA	
*SmActin*-F	GGTGCCCTGAGGTCCTGTT	
*SmActin*-R	AGGAACCACCGATCCAGACA	

**Table A2:** The ANS protein sequence for constructing phylogenetic tree.

ANS Protein	Species Names	GenBank ID
SmANS	*Salvia miltiorrhiza*	MK704422
PsANS	*Paeonia suffruticosa*	AEN71543.1
PrANS	*Paeonia rockii*	AIL29326.1
PlANS	*Paeonia lactiflora*	AFI71900.1
CsANS	*Citrus sinensis*	NP_001275784.1
TcANS	*Theobroma cacao*	ADD51356.1
VvhANS	*Vitis vinifera*	ABV82967.1
FeANS	*Fagopyrum esculentum*	ADT63066.1
FdANS	*Fagopyrum dibotrys*	AHH30830.1
GeANS	*Gypsophila elegans*	AAP13054.1
MiANS	*Matthiola incana*	AAB82287.1
AtANS	*Arabidopsis thaliana*	NP_194019.1
GmANS3	*Glycine max*	AAR26527.1
GmANS2	*Glycine max*	AAR26526.1
GmANS	*Triticum aestivum*	BAE98273.1
GbANS	*Ginkgo biloba*	ACC66093.1
LsANS	*Lactuca sativa*	AVV62510.1
IbANS	*Ipomoea batatas*	BAA75305.1
IcANS	*Ipomoea coccinea*	BAL43066.1
IhANS	*Ipomoea horsfalliae*	ACS71531.1
IpANS	*Ipomoea purpurea*	ABW69684.1
InANS	*Ipomoea nil*	BAB71806.1
IheANS	*Ipomoea hederacea*	AAP82029.1
StANS	*Solanum tuberosum*	NP_001274859.1
NtANS1	*Nicotiana tabacum*	AFM52334.1
NtANS2	*Nicotiana tabacum*	AFM52335.1
ElANS	*Erythranthe lewisii*	AHJ80980.1
SsANS	*Solenostemon scutellarioides*	ABP57081.1
SsANS1	*Solenostemon scutellarioides*	ABP57079.1
DcANS1	*Daucus carota*	AF184273_1
DcANS2	*Daucus carota*	AF184274_1
PpANS	*Prunus persica*	XP_007210520.1
PaANS	*Prunus avium*	ADZ54785.1
PcANS	*Pyrus communis*	AGL50919.1
MdANS	*Malus domestica*	XP_008356542.1
TaANS	*Triticum aestivum*	BAE98273.1
LsANS	*Lactuca sativa*	AVV62510.1
DpANS	*Dahlia pinnata*	BAJ21536.1
RrANS	*Rosa rugosa*	AKT74337.1
OsANS	*Oryza sativa*	CAA69252.1
PgANS	*Punica granatum*	AHZ97874.1
AcANS	*Allium cepa*	ABM66367.1
MtANS	*Medicago truncatula*	ABU40983.1
MaANS	*Morus alba*	AOV62765.1
LcANS	*Lycoris chinensis*	AGD99672.1
LrANS	*Lycoris radiata*	AIA59795.1
OoANS	*Oryza officinalis*	ANY30309.1
ZmaANS	*Zostera marina*	KMZ76142.1
PxhANS	*Petunia x hybrida*	P51092.1
ZmANS	*Zea mays*	NP_001106074.1
